# Outgrowth of Rice Tillers Requires Availability of Glutamine in the Basal Portions of Shoots

**DOI:** 10.1186/s12284-018-0225-2

**Published:** 2018-05-09

**Authors:** Miwa Ohashi, Keiki Ishiyama, Soichi Kojima, Noriyuki Konishi, Kazuhiro Sasaki, Mitsue Miyao, Toshihiko Hayakawa, Tomoyuki Yamaya

**Affiliations:** 1 Graduate School of Agricultural Science, Tohoku University, 468-1 Aoba, Aramaki-Aza, Aoba-ku, Sendai, 980-8572 Japan; 2 The University of Tokyo, Graduate School of Agricultural and Life Sciences, Institute of Sustainable Agro-ecosystem Services (ISAS), 1-1-1 Midori-cho, Nishitokyo, Tokyo, 188-0002 Japan; 3 Present Address: Graduate School of Bioagricultural Sciences, Nagoya University, Furo-cho, Chikusa-ku, Nagoya, 464-8601 Japan; 4 Present Address: Institute of Plant Science and Resources, Okayama University, 2-20-1 Chuo, Kurashiki, 710-0046 Japan; 5 Present Address: Division for Interdisciplinary Advanced Research and Education, Tohoku University, 6-3 Aoba, Aramaki-Aza, Aoba-ku, Sendai, 980-0845 Japan

**Keywords:** Asparagine, Asparagine synthetase, Cytosolic glutamine synthetase, Glutamine, Tiller, Rice

## Abstract

**Background:**

Our previous studies concluded that metabolic disorder in the basal portions of rice shoots caused by a lack of cytosolic glutamine synthetase1;2 (GS1;2) resulted in a severe reduction in the outgrowth of tillers. Rice mutants lacking GS1;2 (*gs1;2* mutants) showed a remarkable reduction in the contents of both glutamine and asparagine in the basal portions of shoots. In the current study, we attempted to reveal the mechanisms for this decrease in asparagine content using rice mutants lacking either GS1;2 or asparagine synthetase 1 (AS1). The contributions of the availability of glutamine and asparagine to the outgrowth of rice tillers were investigated.

**Results:**

Rice has two *AS* genes, and the enzymes catalyse asparagine synthesis from glutamine. In the basal portions of rice shoots, expression of *OsAS1*, the major species in this tissue, was reduced in *gs1;2* mutants, whereas *OsAS2* expression was relatively constant. *OsAS1* was expressed in phloem companion cells of the nodal vascular anastomoses connected to the axillary bud vasculatures in the basal portions of wild-type shoots, whereas cell-specific expression was markedly reduced in *gs1;2* mutants. *OsAS1* was up-regulated significantly by NH_4_^+^ supply in the wild type but not in *gs1;2* mutants. When GS reactions were inhibited by methionine sulfoximine, *OsAS1* was up-regulated by glutamine but not by NH_4_^+^. The rice mutants lacking AS1 (*as1* mutants) showed a decrease in asparagine content in the basal portions of shoots. However, glutamine content and tiller number were less affected by the lack of AS1.

**Conclusion:**

These results indicate that in phloem companion cells of the nodal vascular anastomoses, asparagine synthesis is largely dependent on glutamine or its related metabolite-responsive AS1. Thus, the decrease in glutamine content caused by a lack of GS1;2 is suggested to result in low expression of *OsAS1*, decreasing asparagine content. However, the availability of asparagine generated from AS1 reactions is apparently less effective for the outgrowth of tillers. With respect to the tiller number and the contents of glutamine and asparagine in *gs1;2* and *as1* mutants, the availability of glutamine rather than asparagine in basal portions of rice shoots may be required for the outgrowth of rice tillers.

**Electronic supplementary material:**

The online version of this article (10.1186/s12284-018-0225-2) contains supplementary material, which is available to authorized users.

## Background

Glutamine and asparagine play crucial roles in plant growth and development as the major nitrogen forms for transport via vasculatures from source to sink tissues within various plants (Urquhart and Joy 1981; Ireland and Lea 1999; Lea et al. 2007; Gaufichon et al. 2010). In rice plants, these amides are major nitrogen forms in both phloem (Hayashi and Chino 1990) and xylem sap (Fukumorita and Chino 1982; Funayama et al. 2013; Ohashi et al. 2015a). In higher plants, including rice, glutamine is synthesized by glutamine synthetase (GS) (Lea and Miflin 1974; Yamaya and Oaks 2004), and asparagine is synthesized from glutamine by asparagine synthetase (AS) (Lea et al. 2007; Gaufichon et al. 2010; Ohashi et al. 2015a). Asparagine is catabolized by asparaginase into aspartate as the common precursor of the essential amino acids (Azevedo et al. 2006; Lea et al. 2007; Yabuki et al. 2017).

Rice has three cytosolic GS isoenzymes (GS1, *OsGS1;1* – *OsGS1;3*) and one chloroplastic GS (GS2) (Tabuchi et al. 2007). The occurrence of two AS isoenzymes (*OsAS1* and *OsAS2*) was recently confirmed (Ohashi et al. 2015a). Both GS1;2 and AS1 are important in the primary assimilation of NH_4_^+^ taken up by rice roots (Funayama et al. 2013; Ohashi et al. 2015a). *OsGS1;2* and *OsAS1* were specifically accumulated in three cell layers of root surfaces (epidermis, exodermis and sclerenchyma) after NH_4_^+^ supply to rice roots (Ishiyama et al. 2004; Ohashi et al. 2015a). In both roots and xylem sap after NH_4_^+^ supply, rice mutants lacking GS1;2 showed a decrease in glutamine and asparagine content, while mutants lacking AS1 showed a decrease in asparagine content (Funayama et al. 2013; Ohashi et al. 2015a).

Tiller number is a critical agronomic trait defining grain yields in rice and is influenced by the availability of nitrogen (Mae 1997; Sakamoto and Matsuoka 2008; Liu et al. 2011). We found that the lack of GS1;2 suppressed the outgrowth of the tiller axillary bud and hence a substantial decrease in active tiller number and yields (Funayama et al. 2013; Ohashi et al. 2015b). The outgrowth of tiller axillary buds has been proposed to be related to metabolite use efficiency and hormone signalling networks (Domagalska and Leyser 2011; Evers et al. 2011). We recently showed that metabolic disorder in the basal portions of rice shoots lacking GS1;2 caused a severe reduction in the outgrowth of tiller axillary buds, and this reduction was independent of the content of strigolactone (Ohashi et al. 2015b), a phytohormone inhibiting tiller development (Umehara et al. 2008). The basal portions of rice shoots are important organs for the circulation of metabolites because of vascular networks (Hoshikawa 1989). These organs consist of axillary buds, internodes, and shoot apical meristems (SAMs) (Hoshikawa 1989; Ohashi et al. 2015b). In the basal portions of rice shoots, GS1;2 protein localizes in phloem companion cells of the nodal vascular anastomoses connecting to the axillary bud vasculatures (Ohashi et al. 2015b). The lack of GS1;2 caused a large decrease in both glutamine and asparagine contents in roots and the basal portions of shoots (Ohashi et al. 2017). This decrease in glutamine content in the basal portions of shoots caused a deficiency of active cytokinin, which is required for the outgrowth of rice tillers, via the down-regulation of glutamine or its related metabolite-dependent cytokinin synthesis (Ohashi et al. 2017). Thus, the availability of glutamine and/or asparagine is required for the outgrowth of rice tillers (Ohashi et al. 2015b, 2017).

In the present study, we focused on the reduction of asparagine content in the basal portions of rice mutants lacking GS1;2 (*gs1;2* mutants). Using rice mutants lacking either GS1;2 or AS1 (*as1* mutants), we investigated (1) the molecular mechanisms involved in the decrease in asparagine content in the basal portions of *gs1;2* shoots and (2) the relation between low availability of asparagine and the reduction in tiller number.

## Results

### Reduced Expression of *OsAS1* in Phloem Companion Cells of the Nodal Vascular Anastomoses in the Basal Portions of Shoots Lacking GS1;2

**Fig. 1:**
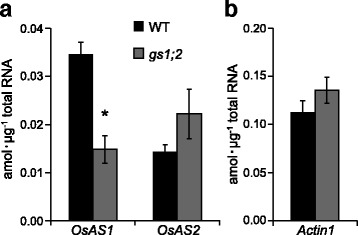
qPCR analysis of *OsASs* in the basal portions of wild-type and *gs1;2* mutant shoots. qPCR analysis of *OsAS1* and *OsAS2* genes (**a**) and a control *Actin1* (**b**) in the basal portions of shoots of wild-type plants (WT: black column) and *gs1;2* mutants (*gs1;2*: grey column) were performed. Rice seedlings were grown hydroponically in the presence of 1 mM NH_4_Cl until the fourth-leaf stage. Mean values with SE of four independent samples are shown. An asterisk denotes a statistically significant difference between wild type and *gs1;2* mutants (*, *P* < 0.05 by Student’s *t*-test)

Expression of *OsAS1* in the basal portions of shoots of NH_4_^+^-fed *gs1;2* mutant seedlings at the fourth-leaf stage showed an approximately 60% reduction compared with the level in the wild-type rice (Fig. 1). However, the expression of *OsAS2* in the *gs1;2* mutants was less affected in the basal portions (Fig. 1). *OsAS1* was expressed at twice the level of *OsAS2* in the basal portions of wild-type shoots (Fig. 1).

**Fig. 2:**
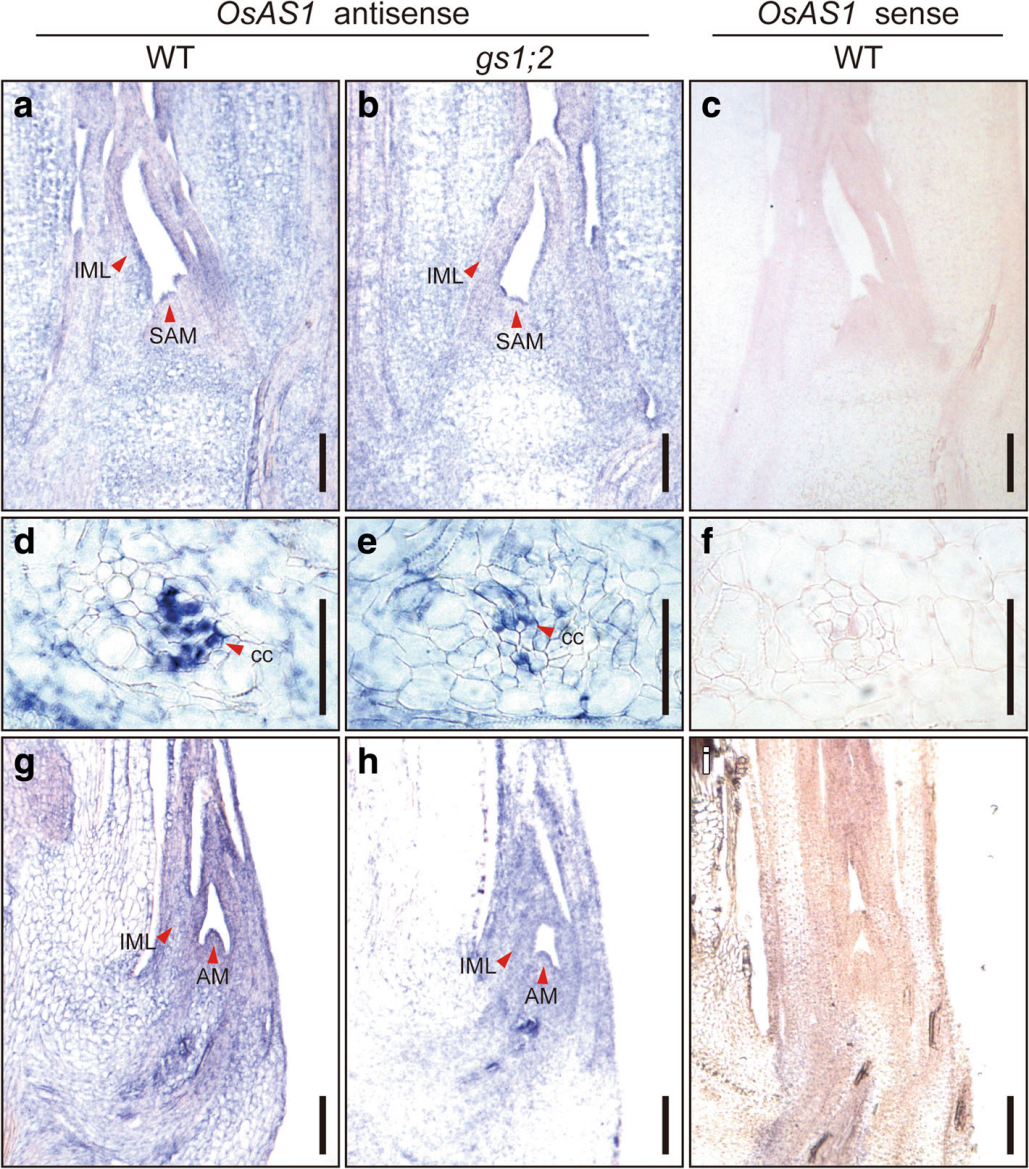
In situ hybridization for *OsAS1* in the basal portions of wild-type and *gs1;2* mutant shoots. Longitudinal sections of basal portions of shoots were prepared from the wild-type rice (WT) (**a**, **c**, **d**, **f**, **g**, **i**) and from *gs1;2* mutants (*gs1;2*) (**b**, **e**, **h**) grown hydroponically in the presence of 1 mM NH_4_Cl until the fourth-leaf stage. A sense probe was hybridized to WT sections (**c**, **f**, **i**) as a negative control. Red arrowheads indicate hybridization signals for *OsAS1* transcripts in the shoot apical meristem (SAM) (**a**, **b**), phloem companion cell of the nodal vascular anastomoses (cc) (**d**, **e**), an immature leaf (IML) area (**a**, **b**, **g**, **h**), and the tiller containing the axillary bud meristem (AM) (**g**, **h**). Note that the signal intensity in *gs1;2* (**e**) was far weaker than in the WT (**d**). Scale bars = 100 μm (**a** - **c**, **g** - **i**) and 50 μm (**d** - **f**)

**Fig. 3:**
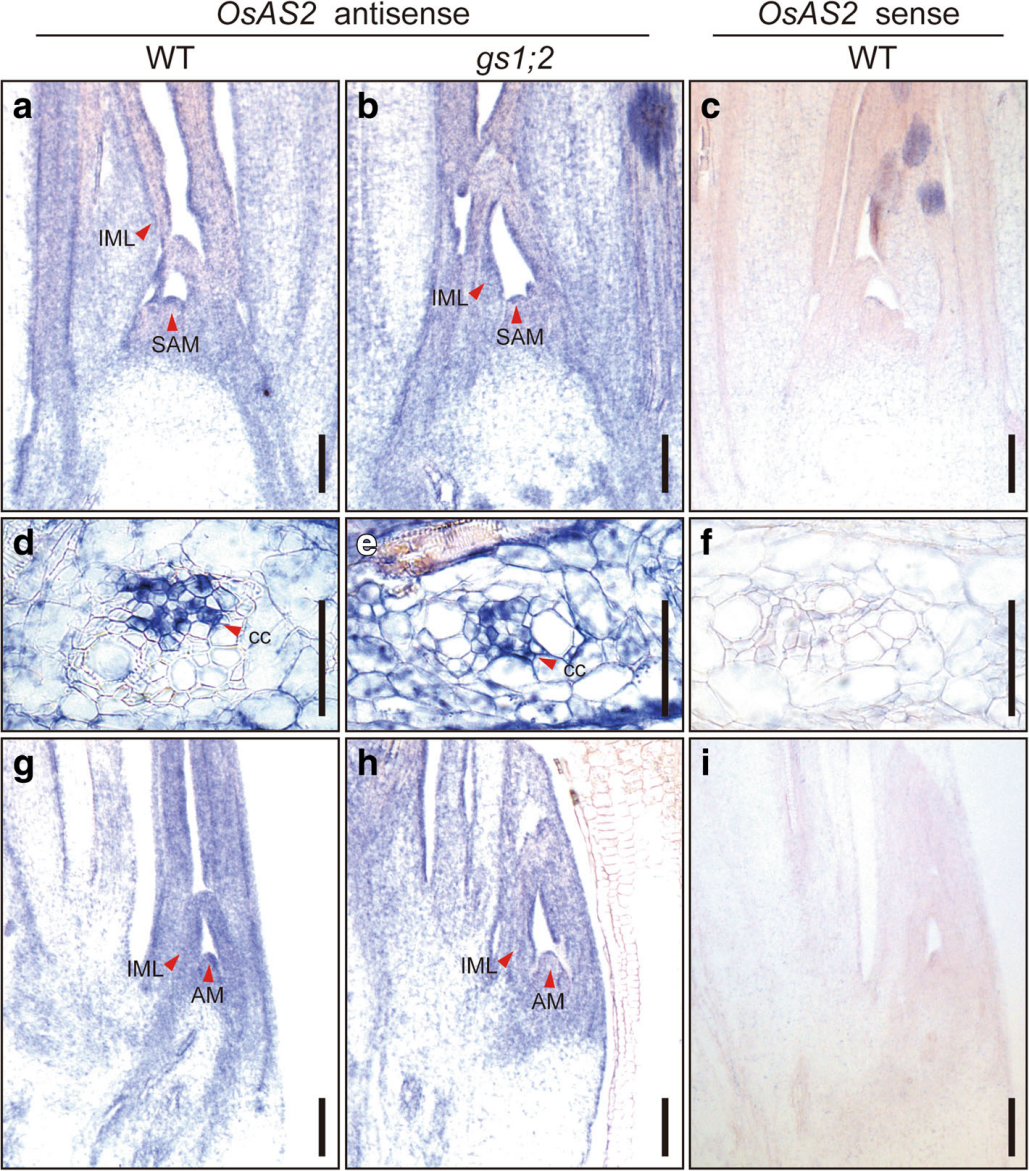
In situ hybridization for *OsAS2* in the basal portions of wild-type and *gs1;2* mutant shoots. Longitudinal sections of basal portions of shoots were prepared from the wild-type rice (WT) (**a**, **c**, **d**, **f**, **g**, **i**) and the *gs1;2* mutants (*gs1;2*) (**b**, **e**, **h**) grown hydroponically in the presence of 1 mM NH_4_Cl until the fourth-leaf stage. A sense probe was hybridized to WT sections (**c**, **f**, **i**) as a negative control. Red arrowheads indicate hybridization signals for *OsAS2* transcripts in the shoot apical meristem (SAM) (**a**, **b**), phloem companion cell of the nodal vascular anastomoses (cc) (**d**, **e**), an immature leaf (IML) area (**a**, **b**, **g**, **h**), and the tiller containing the axillary bud meristem (AM) (**g**, **h**). Scale bars = 100 μm (**a** - **c**, **g** - **i**) and 50 μm (**d** - **f**)

In situ hybridization revealed strong signals for the *OsAS1* transcript in phloem companion cells of the nodal vascular anastomoses in the basal portion of wild-type shoots (Fig. 2d, e). However, the signal intensity for *OsAS1* in phloem companion cells of the nodal vascular anastomoses in the *gs1;2* mutants was much lower than in the wild-type rice. Weak signals for *OsAS1* were also detected in the SAMs and immature leaves in both the *gs1;2* mutants and wild-type rice (Fig. 2a, b, g, h). Signals for the *OsAS2* transcript were also detected in phloem companion cells of the nodal vascular anastomoses, and weak signals were detected in the SAM and immature leaves in both the *gs1;2* mutants and wild-type rice. Unlike *OsAS1,* the signal intensity of the *OsAS2* transcript was not significantly different between *gs1;2* mutants and wild-type rice (Fig. 3a, b, d, e, g, h). These results fit well with the results obtained from quantitative real-time PCR (qPCR) analysis (Fig. 1). When the control sense probes were used, only background levels of the *OsAS1* and *OsAS2* transcript were detected (Figs. 2c, f, i, 3c, f, i).

### Reduction in *OsAS1* Expression and Asparagine Content after NH_4_^+^ Supply to the *gs1;2* Mutants

**Fig. 4:**
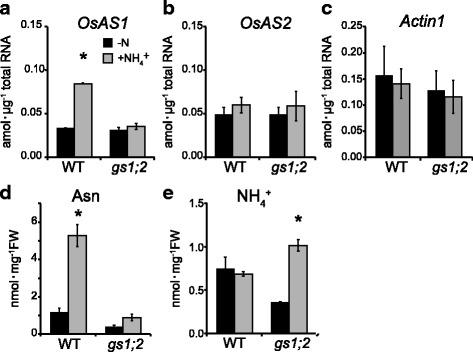
Expression levels of *OsASs* and contents of asparagine and NH_4_^+^ in the basal portions. **a** - **c** Transcript contents of the *OsAS1* (**a**), *OsAS2* (**b**) and a control *Actin1* (**c**) in the basal portions of shoots of wild-type plants (WT: black column) and *gs1;2* mutants (*gs1;2*: grey column) at the seventh-leaf stage for seedlings grown in water for 3 d followed by treatments with (+NH_4_^+^) or without (-N) 1 mM NH_4_Cl for 8 h. (**d**, **e**) Contents of asparagine (**d**) and NH_4_^+^ (**e**) in the basal portions of shoots of WT (black column) and *gs1;2* (grey column) at the fourth-leaf stage for seedlings grown in water for 3 d followed by treatments with or without 1 mM NH_4_Cl for 24 h. Mean values with SE of four independent samples are shown. Asterisks denote statistically significant differences between samples treated with and without 1 mM NH_4_Cl (*, *P* < 0.05 by Student’s *t*-test)

In rice roots, up-regulation of *OsAS1* expression and accumulation of asparagine content were observed after NH_4_^+^ supply (Ohashi et al. 2015a). In the current study, *OsAS1* expression and asparagine content after NH_4_^+^ supply were determined in the basal portions of shoots using *gs1;2* mutant and wild-type plants. *OsAS1* expression was increased over twofold at 8 h after NH_4_^+^ supply in the shoot basal portions of the wild type, whereas it was less changed in the *gs1;2* mutants (Fig. 4a). In contrast, there were no significant differences in the expression of *OsAS2* and *Actin1* between the shoot basal portions of wild type and *gs1;2* mutants treated with or without NH_4_^+^ (Fig. 4b, c). Asparagine was accumulated remarkably at 24 h after NH_4_^+^ supply in the shoot basal portions of the wild type, whereas it was relatively constant in the *gs1;2* mutants (Fig. 4d). NH_4_^+^ was highly accumulated in these tissues in *gs1;2* mutants after NH_4_^+^ supply (Fig. 4e), indicating impairment of NH_4_^+^ assimilation. Under the same conditions, glutamine content was less affected 24 h after NH_4_^+^ supply in the shoot basal portions of *gs1;2* mutants (Ohashi et al. 2017), as in the case of asparagine content in the current study (Fig. 4d).

**Fig. 5:**
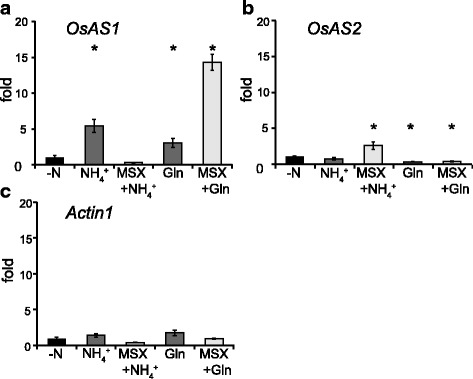
qPCR analysis of *OsASs* in the basal portions of wild-type shoots using MSX. Wild-type rice seedlings were grown hydroponically in the presence of 1 mM NH_4_Cl until the fourth-leaf stage and then transferred into water for 3 d. After pre-treatment with 1 mM MSX for 2 h, the seedlings were treated for 8 h with 1 mM NH_4_^+^ (MSX + NH_4_^+^) or 5 mM glutamine (MSX + Gln). The seedlings without MSX pre-treatment were also treated for 8 h with 1 mM NH_4_^+^ (+NH_4_^+^), 5 mM glutamine (Gln) or without nitrogen nutrients (-N). qPCR analyses of *OsAS1* (**a**), *OsAS2* (**b**), and *actin1* transcripts (**c**) were performed in the shoot basal parts. Fold changes of transcript levels in each treatment relative to those in –N were calculated, and mean values with SE of four independent samples are shown. Asterisks denote statistically significant differences between the samples treated without nitrogen nutrients (-N) and each treated sample (*, *P* < 0.05 by Student’s *t*-test)

Further qPCR analysis was carried out in the presence or absence of 1 mM methionine sulfoximine (MSX), an inhibitor of GS reactions. As shown in Fig. 4a, a substantial increase in *OsAS1* expression was observed at 8 h after NH_4_^+^ supply in the basal portions of shoots, but this increase was not observed when MSX was treated prior to NH_4_^+^ supply (Fig. 5a). Over a three-fold increase in *OsAS1* expression was observed after glutamine supply with or without MSX pre-treatment (Fig. 5a). As in *OsAS1* expression, the significant increase in *OsGS1;2* expression was observed after NH_4_^+^ or glutamine supply, but an increase in *OsGS1;2* expression was not observed for the MSX treatment (Additional file 1: Figure S1b). The expression of *OsAS2* was stable after NH_4_^+^ supply, while glutamine supply caused a decrease in its expression (Fig. 5b). There were slight fluctuations in the expression of *OsGS1;1* and *Actin1* used as a control treated with or without NH_4_^+^ (Fig. 5c, Additional file 1: Figure S1a).

In the roots, *OsAS1* expression was increased significantly after NH_4_^+^ supply, but this increase was not observed in the MSX treatment (Additional file 2: Figure S2a). Increases in *OsAS1* expression were observed after glutamine supply with or without MSX treatment (Additional file 2: Figure S2a).

### Lack of AS1 Caused a Reduction in Asparagine Content in the Basal Portions of Rice Shoots but not in Tiller Number

**Fig. 6:**
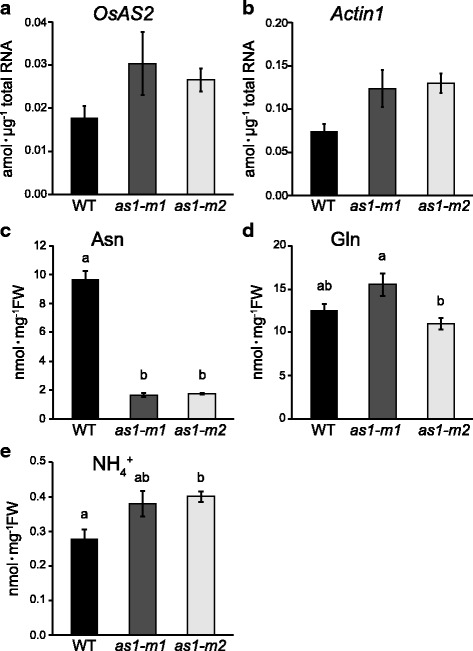
qPCR and amino acid analysis in the basal portions of wild-type and *as1* mutant shoots. qPCR analysis of *OsAS2* (**a**) and *Actin1* (**b**) and analyses of asparagine (**c**), glutamine (**d**) and NH_4_^+^ contents (**e**) were performed in the basal portions of shoots of the wild type (WT: black column) and two lines of *as1* mutants (*as1-m1* and *as1-m2*: dark and light grey column, respectively). Seedlings were grown hydroponically in the presence of 1 mM NH_4_Cl until the fourth-leaf stage. Mean values with SE of four independent samples are shown. One-way ANOVAs followed by Bonferroni tests were used to identify significant differences between the wild type and *as1* mutants (*P* < 0.05)

To examine the contribution of availability of asparagine generated from AS1 reactions in the basal portions of shoots to the outgrowth of rice tillers, reverse genetics studies were carried out using homozygous rice mutants lacking *OsAS1* (Ohashi et al. 2015a). Our previous study showed that the lack of AS1 did not affect the expression of other genes related to nitrogen metabolism in roots (Ohashi et al. 2015a). In the basal portions of shoots growing with NH_4_^+^, the *as1* mutants exhibited no significant difference in *OsAS2* expression, but an approximately 80% decrease in asparagine content was observed compared with the content in the wild type (Fig. 6a, c). In addition, transient NH_4_^+^ supply for 24 h to nitrogen-depleted *as1* mutants caused an approximately 60% decrease in asparagine content in the shoot basal portions (Additional file 3: Figure S3a). There were slight fluctuations in the content of glutamine and NH_4_^+^ between the basal portions of shoots of *as1* mutants and the wild type when plants were grown with NH_4_^+^ (Fig. 6d, c, Additional file 3: Figure S3b, d).

**Fig. 7:**
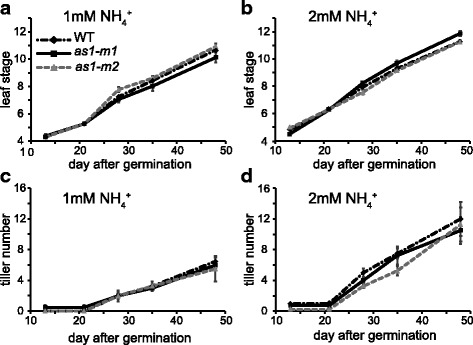
Comparison of leaf stage and tiller number between the wild type and *as1* mutants. Wild-type (WT: black diamond) plants and two lines of *as1* mutants (*as1-m1* and *as1-m2*: black square and grey triangle, respectively) were grown hydroponically in the presence of 1 mM (**a**, **c**) or 2 mM NH_4_Cl (**b**, **d**) until 50 days after germination. Time course studies on measurements of leaf stage and tiller number were conducted. Mean values with SE of four independent samples are shown. One-way ANOVAs followed by Bonferroni tests were used to identify significant differences between the wild type and *as1* mutants (*P* < 0.05)

No significant differences between *as1* mutants and the wild type were observed with respect to tiller number and leaf stage under 1 mM and 2 mM NH_4_^+^ (Fig. 7). The outgrowth of primary tiller was little affected in *as1* mutants grown with 1 mM NH_4_^+^ until fifth leaf stage (Additional file 4: Figure S4). The same is true when those plants were grown in the paddy field under normal fertilization (Table 1). There were also no significant differences in shoot length, dry weight, or panicle number between *as1* mutants and wild-type plants grown in the paddy field (Table 1). In our experiments in the paddy field, the lodging phenotype was not observed in *as1* mutants (Additional file 5: Figure S5).

**Table 1:** Tiller number, panicle number and parameters of growth of plants growing in the paddy field

	wild type	*as1-m1*	*as1-m2*
Tiller number per plant	25.7 ± 1.83	25.35 ± 1.01	22.35 ± 0.49
	a	a	a
Panicle number per plant	20.32 ± 1.19	20.55 ± 0.71	18.66 ± 0.98
	a	a	a
Shoot length (cm)	100.9 ± 2.23	100.65 ± 1.62	94.1 ± 0.99
	a	a	a
Shoot dry weight per plant (g)	57.69 ± 4.47	65.35 ± 1.79	50.76 ± 3.13
	ab	a	b

Wild-type plants and two lines of *as1* mutants (*as1-m1* and *as1-m2*) were grown in the paddy field under normal fertilization. Tiller number was measured at the panicle initiation stage. After panicle ripening, rice plants were harvested and shoot length, shoot dry weight without panicle and panicle number were measured. Mean values with SE of four independent plots (five average plants selected from each plot) are shown. One-way ANOVA followed by Bonferroni tests were used to identify significant differences between the wild type and *as1* mutants (*P* < 0.05).

## Discussion

A lack of GS1;2 caused a reduction in asparagine content in the basal portions of shoots (Ohashi et al. 2017). The current study showed that the expression of *OsAS1*, the major species in the basal portions of shoots, was decreased by the lack of GS1;2, whereas *OsAS2*, which is dominant in rice leaves, was less affected (Fig. 1) (Ohashi et al. 2015a). In fact, the lack of AS1 severely decreased the asparagine content in the basal portions of shoots (Fig. 6c). These results indicate that the low expression of *OsAS1* caused a decrease in asparagine content in the shoot basal portions of *gs1;2* mutants. In the basal portions of shoots, GS1;2 and AS1 are suggested to be important in the biosynthesis of asparagine, whereas the contribution of AS2 to this process may be limited (Fig. 1).

Gene expression analyses clearly showed that the expression of *OsAS1*, but not *OsAS2*, in both roots and shoot basal portions was enhanced by NH_4_^+^ and glutamine (Figs. 4, 5, Additional file 2: Figure S2) (Ohashi et al. 2015a). The NH_4_^+^-induced *OsAS1* expression was inhibited when GS1;2 was missing or GS activity was inhibited by MSX treatment (Figs. 4, 5, Additional file 2: Figure S2). In addition, *OsAS1* expression was enhanced by glutamine when GS activity was inhibited by MSX treatment. These results are in close agreement with a previous study by Kawachi et al. (2002), which showed that the NH_4_^+^-induced accumulation of AS protein in rice roots was completely inhibited by MSX treatment. These results indicate that glutamine or its related metabolite, but not NH_4_^+^ itself, acts as a signal molecule for the stimulation of *OsAS1* expression in both roots and shoot basal portions, where AS1 is the major isoform (Fig. 5, Additional file 2: Figure S2) (Ohashi et al. 2015a). In addition, the expression of *OsAS1* in phloem companion cells of nodal vascular anastomoses, overlapping with *OsGS1;2* expression (Ohashi et al. 2015b), was reduced by the lack of GS1;2 (Fig. 2d, e). These results possibly allow glutamine, which is produced by GS1;2, to directly induce the expression of *OsAS1* and to be used for asparagine synthesis within these cells. We conclude that the low availability of glutamine caused reduced expression of *OsAS1*, decreasing asparagine synthesis, in phloem companion cells of the nodal vascular anastomoses in the basal portions of shoots in the *gs1;2* mutants.

There are also several genes reported to be up-regulated by glutamine or related metabolites in rice and other plants, i.e., in rice, *ammonium transporter1;1* (*OsAMT1;1*) and *OsAMT1;2* (Sonoda et al. 2003), *NADH-glutamate synthase1* (Hirose et al. 1997), and *adenosine phosphate-isopentenyltransferases4* (*IPT4*) and *IPT5* (Kamada-Nobusada et al. 2013); in Arabidopsis, *glutamine symtnetase1* (Oliveira and Coruzzi 1999), *AtAMT1;1* (Rawat et al. 1999), and *AtAS* (Lam et al. 1994); and nitrate reductase in tobacco leaves (Vincentz et al. 1993). The signalling mechanism in response to glutamine apparently contributes to modulating plant growth. However, few details are known regarding glutamine signalling, unlike the case of nitrate signalling (Sakakibara et al. 2006). It is worth examining the glutamine responsive induction of *OsAS1* to understand the mechanism of glutamine signalling in response to NH_4_^+^.

As discussed previously (Ohashi et al. 2015b), GS1;2 has a role in the re-assimilation of NH_4_^+^ released from the phenylalanine ammonia-lyase (PAL) reaction during lignification in the basal portions of shoots. In fact, the biosynthesis of lignin accounts for 10–23% in rice internodes (Ookawa and Ishihara 1993; Ookawa et al. 1993), and PAL activity in the basal portions of rice shoots was approximately 20-fold higher than in leaf blade at the seedling stage (Additional file 6: Figure S6). Thus, in the basal portions of rice shoots, NH_4_^+^ released during lignification must be re-assimilated immediately to avoid the potential toxicity of NH_4_^+^ and the loss of nitrogen (Sakurai et al. 2001). In the basal portions of shoots, AS1 and GS1;2 could contribute to the re-assimilation of NH_4_^+^ during lignification. Considering its cellular localization in phloem companion cells of the nodal vascular anastomoses, the asparagine from AS1 reactions in these cells is apparently transported to sink organs, such as axillary buds, for its outgrowth and development (Fig. 2). Asparagine is catabolized by asparaginase into aspartate as the common precursor of the essential amino acids (Azevedo et al. 2006; Lea et al. 2007; Yabuki et al. 2017).

The lack of GS1;2 caused decreases in tiller number, rice yields and glutamine and asparagine content in the basal portions of shoots (Funayama et al. 2013; Ohashi et al. 2015b, 2017), while a lack of AS1 caused a decrease in only asparagine content and had less of an effect on tiller number and glutamine content (Figs. 6, 7, Table 1). These results indicate that the availability of asparagine generated from AS1 reactions apparently contributes less to the outgrowth of tillers in rice plants under conditions of sufficient glutamine. Thus, we conclude that the reduction in tiller number in *gs1;2* mutants is independent of the low availability of asparagine in the basal portions of shoots. Previous research has revealed that glutamine generated from GS1;2 reactions contributes to the outgrowth of tillers via glutamine-dependent cytokinin synthesis (Ohashi et al. 2017). Compared to that of glutamine, the contribution of asparagine to the outgrowth of rice tillers is suggested to be limited.

## Conclusion

Our results demonstrated that the expression of *OsAS1* could be induced by glutamine or its related metabolite in both roots and the basal portions of rice shoots. Especially in phloem companion cells of the nodal vascular anastomoses, asparagine synthesis is largely dependent on glutamine-responsive AS1. Thus, we conclude that the low availability of asparagine in *gs1;2* mutants was caused by the reduction in glutamine content, which is required for the up-regulation of *OsAS1* expression. Availability of glutamine is suggested to be more important than that of asparagine for the outgrowth of rice tillers.

## Methods

### Plant Materials

This study utilized the rice (*Oryza. sativa* L.) cultivar ‘Nipponbare’ as the wild-type plants as well as a retrotransposon *Tos17*-inserted line of the *gs1;2* mutant and two lines of *as1* mutants (Funayama et al. 2013; Ohashi et al. 2015a). The seeds of these rice plants were germinated and grown hydroponically until either the fourth- or seventh-leaf stage in an outdoor greenhouse. The temperature was controlled at 26 °C during the day with supplemental light for 13 h. The hydroponic culture solution was renewed once per week with 1 mM NH_4_Cl as described in Ohashi et al. (2017). Longitudinal sections (5 mm) of basal portions of shoots including axillary buds, internodes and a SAMs were prepared by removing the primary and secondary leaves, seeds and roots as described in Ohashi et al. (2017). The basal portions of shoots at the fourth-leaf stage or seventh-leaf stage were used for the determination of the amino acid and NH_4_^+^ content, in situ hybridization, and qPCR analyses.

When the short-term effects of NH_4_^+^ supply to roots on gene expression and determination of the free amino acid and NH_4_^+^ content were tested, the seedlings at the fourth-leaf stage or seventh-leaf stage were further grown in water for 3 d to deplete nitrogen and were then treated with or without 1 mM NH_4_Cl for 8 h or 24 h (Ohashi et al. 2017). In MSX-treatment experiments, the seedlings at the fourth-leaf stage grown in water for 3 d were pretreated with or without 1 mM MSX (Sigma-Aldrich Japan, Tokyo, Japan) for 2 h and then transferred to media containing no nitrogen, 1 mM NH_4_C1 or 5 mM glutamine for 8 h according to Kamada-Nobusada et al. (2013) with slight modifications.

For measuring the tiller number, panicle number, shoot length and shoot dry weight at a paddy field, rice plants were grown in the paddy field of the Institute for Sustainable Agro-ecosystem Services in University of Tokyo, Japan (35°44’ N, 139°32′ E, 60 m altitude), during the summer (May to October) in 2015 under normal fertilizer, light irradiance and temperature. A germinated seed was sown into each cell of a cell tray and grown in a greenhouse under natural light conditions for four weeks. One seedling was then transplanted into each hill at 18 cm between hills and 30 cm between rows. Each plot was composed of four rows with ten hills with four replicates. Commercial compound fertilizer including N, P_2_O_5_ and K_2_O was used to fertilize at 60, 90, and 80 kg ha^− 1^ as a basal fertilizer before transplanting. At the panicle initiation stage, tiller numbers of five average plants selected from each plot were measured. After panicle ripening, ten plants in each plot were harvested and shoot length, shoot dry weight without panicle, and panicle number were measured.

### qPCR Analysis

The qPCR analysis was performed according to Konishi et al. (2014). The nucleotide sequences for *OsAS1*, *OsAS2*, *OsGS1;1*, *OsGS1;2* and *Actin1* are registered in the Rice Annotation Project Database (RAP-DB; http://rapdb.dna.affrc.go.jp/) with accession numbers Os03g0291500, Os06g0265000, Os02g0735200, Os03g0223400 and Os03g0718100, respectively. Each gene-specific primer for qPCR analyses is shown in Tabuchi et al. (2007) and Ohashi et al. (2015a). The PCR products were amplified from the single-stranded cDNA as the template and quantified using a Light Cycler 480 (Roche Diagnostics K.K., Tokyo, Japan) according to the following program: 10 s at 95 °C, followed by 50 cycles of 95 °C for 5 s and 60 °C for 34 s. The transcript contents were quantitatively determined using each purified cDNA clone as a calibration standard.

### In situ Hybridization of *OsAS* Genes

Preparation of RNA probes for *OsAS1* and *OsAS2* and in situ hybridization analysis for the basal portions of shoots were performed as described in Ohashi et al. (2015a, 2017).

### Determination of Glutamine, Asparagine and NH_4_^+^ Contents

Free amino acids and NH_4_^+^ contents in the basal portions of shoots at the fourth-leaf stage were determined as described in Konishi et al. (2014). Derivatization of amino acids and NH_4_^+^ was carried out using the AccQ-Tag Ultra Derivatization Kit (Nihon Waters K.K., Tokyo, Japan). AccQ-Tag-labelled derivatives were separated and quantified using an ACQUITY UPLC H-Class with a tunable UV detector (Nihon Waters K.K.).

### Analysis of Phenylalanine Ammonia-lyase Activity

The assay for PAL activity was conducted according to Olsen et al. (2008) with slight modifications. Shoot basal portion tissues (80–170 mg fresh weight) were pre-chilled in liquid nitrogen and then milled with a Tissue Lyser II (Qiagen, K. K., Tokyo, Japan) at 20 Hz for 2 min. Powdered samples were resolved in 5 volumes of extraction buffer (100 mM Tris–HCl (pH 8.8) with 12 mM β-mercaptoethanol) of sample weight, mixed in Tissue Lyser II at 20 Hz for 2 min, and centrifuged at 16,000 g for 15 min at 4 °C. The supernatant was passed through a MicroSpin S-200 HR Column (GE Healthcare Japan, Tokyo, Japan), and the elution as enzyme solution was used for the PAL activity assay. The PAL activity assay was performed at 37 °C for 60 min in an assay mixture containing 500 μl enzyme extract, 450 μl 100 mM Tris–HCl (pH 8.8) and 50 μl 100 mM L-phenylalanine. The reaction was terminated by adding 50 μl of 5 M HCl, and absorbance was recorded at 290 nm against blanks made in the same way as the assays but with 50 μl of 5 M HCl added before L-phenylalanine. The amount of product formed was calculated from the increase in absorbance using an extinction coefficient for cinnamate of 10,000 L/cm/mole (Zimmerman and Hahlbrock 1975).

### Statistics

All data sets were analysed using Microsoft Excel add-in software (Social Survey Research Information Co., Ltd., Tokyo, Japan).

## Additional files

Additional file 1: **Figure S1.** qPCR analysis of *OsGS1;1* and *OsGS1;2* in the basal portions of wild-type shoots using MSX. Wild-type rice seedlings were grown hydroponically in the presence of 1 mM NH_4_Cl until the fourth-leaf stage and then in water for 3 d. After pre-treatment with 1 mM MSX for 2 h, the seedlings were treated for 8 h with 1 mM NH_4_^+^ (MSX + NH_4_^+^) or 5 mM glutamine (MSX + Gln). The seedlings without MSX pre-treatment were also treated for 8 h with 1 mM NH_4_^+^ (+NH_4_^+^), 5 mM glutamine (Gln) or without nitrogen nutrients (-N). qPCR analysis of *OsGS1;1* (a) and *OsGS1;2* (b) were performed in the shoot basal parts. Fold changes of transcript levels in each treatment relative to those in –N were calculated, and mean values with SE of four independent samples are shown. Asterisks denote statistically significant differences between samples treated without 1 mM NH_4_^+^ and each treated sample (*, *P* < 0.05 by Student’s *t*-test). (EPS 1807 kb)

Additional file 2: **Figure S2.** qPCR analysis of *OsAS1* and *OsAS2* in roots of wild-type rice using MSX. Wild-type rice seedlings were grown hydroponically in the presence of 1 mM NH_4_Cl until the fourth-leaf stage and then transferred into water for 3 d. After pre-treatment with 1 mM MSX for 2 h, the seedlings were treated for 8 h with 1 mM NH_4_^+^ (MSX + NH_4_^+^) or 5 mM glutamine (MSX + Gln). The seedlings without MSX pre-treatment were also treated for 8 h with 1 mM NH_4_^+^ (+NH_4_^+^), 5 mM glutamine (Gln) or without nitrogen nutrients (-N). qPCR analyses of *OsAS1* (a), *OsAS2* (b), and *actin1* transcripts (c) were performed in roots. Fold changes of transcript levels in each treatment relative to those in –N were calculated, and mean values with SE of four independent samples are shown. Asterisks denote statistically significant differences between samples treated without 1 mM NH_4_^+^ and each treated sample (*, *P* < 0.05 by Student’s *t*-test). (EPS 1890 kb)

Additional file 3: **Figure S3.** Amino acid analysis in the basal portions of shoots supplied with transient NH_4_^+^. Seedlings of the wild type (WT: black column) and two lines of *as1* mutants (*as1-m1* and *as1-m2*: dark and light grey column, respectively) at the fourth-leaf stage were grown in water for 3 d, then treated with (+NH_4_^+^) or without (-N) 1 mM NH_4_Cl for 24 h. Mean values with SE of four independent samples are shown. One-way ANOVAs followed by Bonferroni tests were used to identify significant differences between the WT and *as1* mutants (*P* < 0.05). (EPS 1925 kb)

Additional file 4: **Figure S4.** Stereoscopic microscope observation of the primary tiller at fifth leaf stage. Seedlings of the wild type (WT) (a) and two lines of *as1* mutants (*as1-m1* and *as1-m2*) (b, c) were grown hydroponically in the presence of 1 mM NH_4_^+^ until the fifth leaf stage. The primary tiller was observed by microscope. Scale bars = 2 mm. (JPG 1217 kb)

Additional file 5: **Figure S5.** Photos of wild type and *as1* mutants at heading stage. Wild-type plants (WT) (a) and two lines of *as1* mutants (*as1-m1* and *as1-m2*) (b, c) were grown in the paddy field under normal fertilization. Each two plants were shown. (JPG 1956 kb)

Additional file 6: **Figure S6.** Comparison of the PAL activity between basal portions of shoots and the third-leaf blade. Rice seedlings were grown hydroponically in the presence of 1 mM NH_4_Cl until the fourth-leaf stage, and the basal portions of shoots (black column) and third-leaf blades (grey column) were harvested. Mean values with SE of five independent samples are shown. Asterisks denote statistically significant differences between each organ (*, *P* < 0.05 by Student’s *t*-test). (EPS 1857 kb)

## Abbreviations

AMT: Ammonium transporter
AS: Asparagine synthetase
GS: Glutamine synthetase
IPT: Adenosine phosphate-isopentenyltransferase
MSX: Methionine sulfoximine
PAL: Phenylalanine ammonia-lyase
qPCR: Quantitative real-time PCR
SAM: Shoot apical meristem
