# Supplementary figures and images for: Outgrowth of Rice Tillers Requires Availability of Glutamine in the Basal Portions of Shoots
Source: Rice (N Y). 2018 May 9;11:31. doi: 10.1186/s12284-018-0225-2 (PMC5943206; doi:10.1186/s12284-018-0225-2)

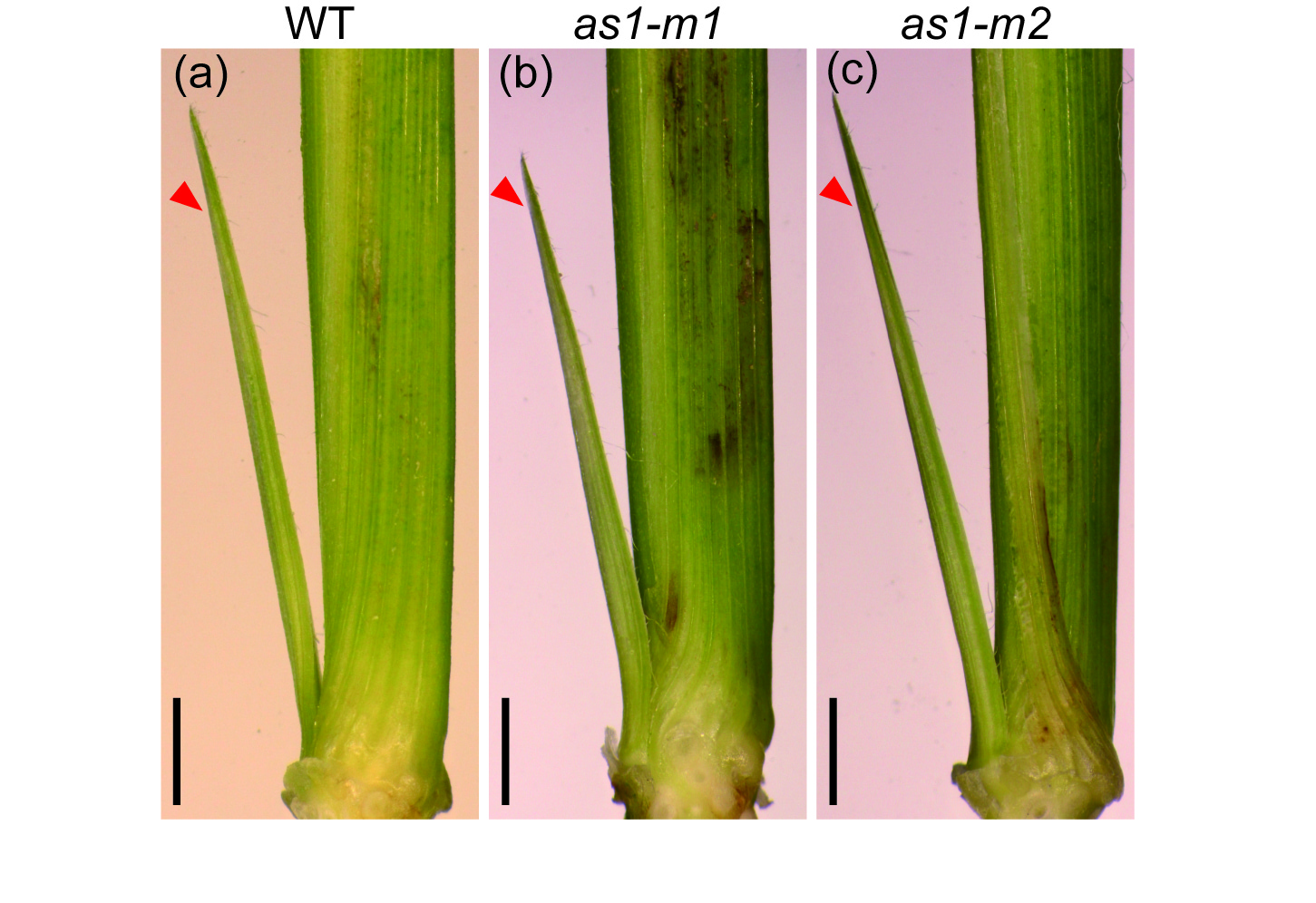

Supplement: Supplementary file 4 — Figure S4. Stereoscopic microscope observation of the primary tiller at fifth leaf stage. Seedlings of the wild type (WT) (a) and two lines of as1 mutants (as1-m1 and as1-m2) (b, c) were grown hydroponically in the presence of 1 mM NH4+ until the fifth leaf stage. The primary tiller was observed by microscope. Scale bars = 2 mm. (JPG 1217 kb) [file 12284_2018_225_MOESM4_ESM.jpg]

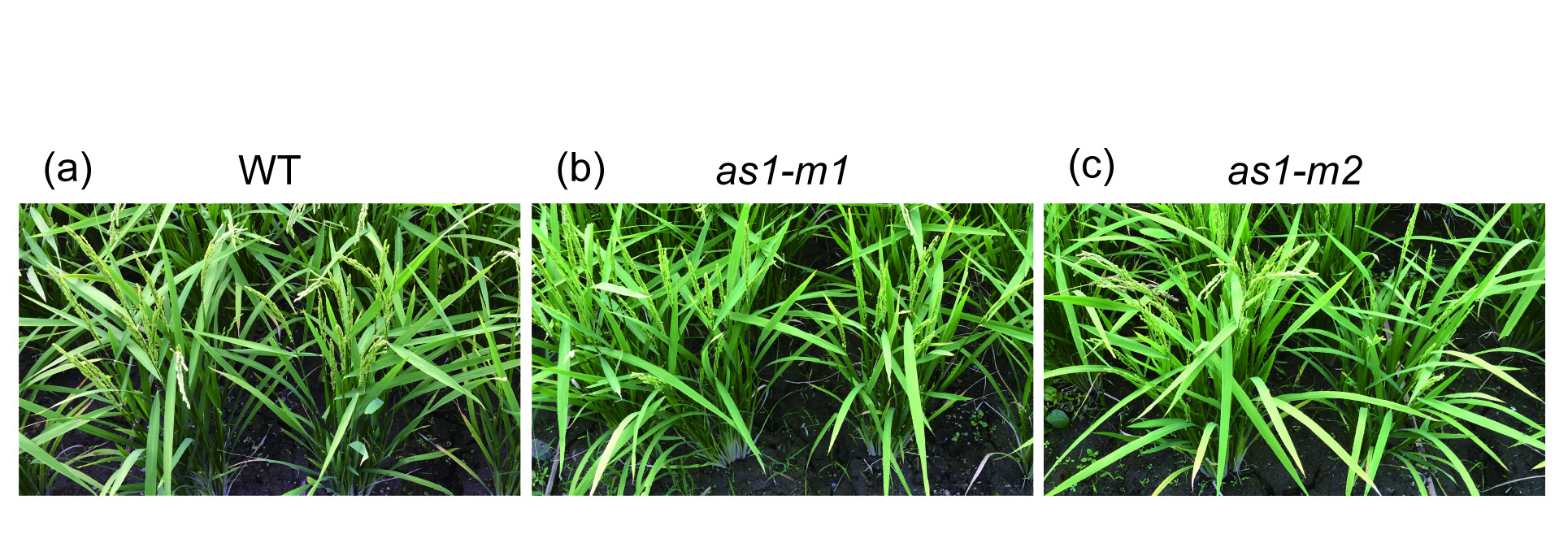

Supplement: Supplementary file 5 — Figure S5. Photos of wild type and as1 mutants at heading stage. Wild-type plants (WT) (a) and two lines of as1 mutants (as1-m1 and as1-m2) (b, c) were grown in the paddy field under normal fertilization. Each two plants were shown. (JPG 1956 kb) [file 12284_2018_225_MOESM5_ESM.jpg]
